# Correlation of bone metabolism with serum pain mediators and inflammatory cytokines in knee osteoarthritis and their role in predicting poor rehabilitation outcomes: A prospective cohort study

**DOI:** 10.5937/jomb0-61488

**Published:** 2026-05-22

**Authors:** Yan Tao, Nan Cai, Juxia Zhang, Yaoping Zhao, Yan Zhou, Pengfei Liu

**Affiliations:** 1 Beijing Jishuitan Hospital, Capital Medical University, Department of Pain Management, Beijing, China; 2 Beijing Jishuitan Hospital, Capital Medical University, Department of Anesthesiology, Beijing, China; 3 Beijing Shijitan Hospital, Capital Medical University, Department of Anesthesiology, Beijing, China

**Keywords:** bone metabolism, knee osteoarthritis, pain mediators, inflammatory factors, diagnostic models, metabolizam kostiju, osteoartritis kolena, medijatori bola, inflamatorni faktori, dijagnostički modeli

## Abstract

**Background:**

The present study was designed to investigate how bone metabolism markers - specifically Procollagen Type I N-Terminal Propeptide (PINP) and C-Telopeptide of Type I Collagen (CTX) - correlate with serum pain mediators (Prostaglandin E2 [PG E2], Norepinephrine [NE], Substance P [SP]) and inflammatory cytokines (Interleukin-1p [IL-ip], Interleukin-6 [IL-6], Tumor Necrosis Factor-a [TNF-a]) in individuals with knee osteoarthritis (KOA). A further aim was to develop a multi-biomarker predictive model for identifying patients at risk of suboptimal rehabilitation outcomes to guide targeted clinical management.

**Methods:**

In this prospective cohort study, 184 KOA patients and an 180 healthy controls were enrolled between January 2023 and May 2024. Using baseline serum, biomarker levels were assessed; Enzyme-Linked Immunosorbent Assay (ELISA) was employed for all analytes except SP, which was determined by radioimmunoassay. The primary endpoint, suboptimal rehabilitation outcome, was determined as either a &lt;30% enhancement in the Western Ontario and McMaster Universities Osteoarthritis Index (WOMAC) score or a &lt;2-point decrease on the Visual Analogue Scale (VAS). Model construction utilized multivariate logistic regression, with its performance evaluated through receiver operating characteristic (ROC) curve analysis, obtaining the area under the curve (AUC), sensitivity, and specificity.

**Results:**

KOA patients exhibited significantly lower serum PINP levels but elevated levels of CTX, PGE2, NE, SP, IL-1p, IL-6, and TNF-a compared to controls (P&lt; 0.05). PINP demonstrated significant inverse correlations with the pain and inflammatory biomarkers, while CTX showed strong positive correlations with them (P&lt; 0.05). The integrative logistic regression model, integrating PINP, CTX, inflammatory cytokines, and pain mediators, demonstrated excellent predictive value for poor outcomes, with an AUC of 0.862, 83.58% sensitivity, and 78.63% specificity (P&lt; 0.05).

**Conclusions:**

Dysregulated bone metabolism (characterized by low PINP and high CTX) in KOA is significantly linked to heightened expression of pain-associated and inflammatory markers. A combined panel of these biomarkers serves as an effective tool for predicting individuals likely to experience suboptimal rehabilitation results.

## Abstract (sr)

**Uvod:**

Ova studija je osmisljena da istraži kako markeri metabolizma kostiju - posebno prokolagen tipa I N-terminalni propeptid (PINP) i C-telopeptid kolagena tipa I (CTX) - koreliraju sa medijatorima bola u serumu (prostaglandin E2 [PGE2], norepinefrin [NE], supstanca P [SP]) i inflamatornim citokinima (interleukin-ip [IL-ip], interleukin-6 [IL-6], faktor tumorske nekroze-a [TNF-a]) kod osoba sa osteoartritisom kolena (KOA). Dalji cilj je bio razvoj prediktivnog modela sa vise biomarkera za identifikaciju pacijenata sa rizikom od suboptimalnih ishoda rehabilitacije kako bi se usmerilo ciljano kliničko lečenje.

**Metode:**

U ovoj prospektivnoj kohortnoj studiji, 184 pacijenta sa osteoartritisom (KOA) i 180 zdravih osoba su uključeni između januara 2023. i maja 2024. godine. Koristeći osnovne kontrole, procenjeni su nivoi biomarkera; enzimski imunosorbentni test (ELISA) je korišćen za sve analite osim SP koji je određen radioimunološkim testom. Primarni krajnji cilj, suboptimalni ishod rehabilitacije, određen je ili kao povećanje rezultata &lt;30% na indeksu osteoartritisa (WOMAC) Univerziteta Zapadni Ontario i Makmaster ili smanjenje &lt;2 poena na vizuelnoj analognoj skali (VAS). Konstrukcija modela je koristila multivarijantnu logističku regresiju, a njene performanse su procenjene analizom ROC krive, dobijajući površinu ispod krive (AUC), osetljivost i specifičnost.

**Rezultati:**

Pacijenti sa KOA pokazali su značajno niže nivoe serumskog PINP-a, ali povišene nivoe CTX, PGE2, NE, SP, IL-1b, IL-6 i TNF-a u poređenju sa kontrolnom grupom (P&lt; 0,05). PINP je pokazao značajne inverzne korelacije sa biomarkerima bola i inflamacije, dok je CTX pokazao jake pozitivne korelacije sa njima (P&lt; 0,05). Model integrativne logističke regresije, koji integriše PINP, CTX, inflamatorne citokine i medijatore bola, pokazao je odličnu prediktivnu vrednost za loše ishode, sa AUC od 0,862, osetljivošću od 83,58% i specifičnošću od 78,63% (P&lt; 0,05).

**Zaključak:**

Disregulisani metabolizam kostiju (karakterisan niskim PINP i visokim CTX) kod KOA je značajno povezan sa ekspresijom markera povezanih sa bolom i inflamatornih markera. Kombinovani panel ovih biomarkera služi kao efikasan alat za predviđanje pojedinaca koji će verovatno imati suboptimalne rezultate rehabilitacije.

## Introduction

Knee osteoarthritis (KOA) ranks among the most prevalent degenerative joint disorders across the globe [1]. According to epidemiological surveys in China, KOA affects 5,016.52/100,000 of the population aged 40 and above, and this percentage is rising due to demographic aging [2]. The key pathological hallmarks of KOA include articular cartilage degeneration, synovial inflammation, and irregular bone remodeling. Clinically, it mainly presents with joint pain, stiffness, and functional impairment— symptoms that severely compromise patients' wellbeing while creating substantial medical and socioeconomic pressures [3]. Despite advancements in understanding KOA's pathogenesis in recent years, the complex »cartilage-bone-synovium« crosstalk has not been fully unraveled. In particular, how abnormal bone metabolism contributes to disease development and the clinical utility of relevant molecular markers are issues that demand further study.

Contemporary studies have verified that disrupted bone metabolism serves as a critical driver of KOA progression [4]. Normally, bone metabolism is balanced through osteoblast-mediated formation and osteoclast-driven resorption. In KOA, however, »imbalanced bone remodeling« predominates, a state where bone resorption is enhanced and bone formation is inhibited. This imbalance gives rise to typical pathological changes, including subchondral bone sclerosis and osteophyte development [5]. Meanwhile, pain mediators and inflammatory cytokines that are highly expressed in the synovium and synovial fluid can trigger pain-signaling neural pathways, accelerate chondrocyte apoptosis, and boost osteoclast activity—ultimately exacerbating joint damage and pain [6]. Nevertheless, the majority of current research focuses on how individual indicators correlate with KOA severity [7][8]. There remains a lack of comprehensive analysis regarding the multi-dimensional interactions among bone metabolism, inflammatory markers, and pain-related mediators. Furthermore, despite attempts to utilize serological biomarkers for predicting KOA progression [9], no dedicated model has been established to forecast unfavorable rehabilitation outcomes, thus restricting the translational value of existing biomarkers.

Drawing on the above-described research status, this study seeks to move beyond the traditional single-dimensional research paradigm. For the first time, it systematically investigates the correlations between bone metabolism parameters, serum pain mediators, and inflammatory cytokines in KOA patients, with the goal of uncovering their pathological connections. Concurrently, by integrating clinical follow-up data, the study assesses how effectively the combined use of these indicators predicts suboptimal rehabilitation outcomes and develops a risk prediction model based on multiple biomarkers. These findings will not only advance the understanding of the "bone metabolism-pain-inflammation" interaction mechanism in KOA but also offer an objective foundation for clinically precise identification of high-risk individuals with suboptimal rehabilitation and the design of personalized intervention strategies.

## Materials and methods

### Study participants and sample size determination

Between January 2023 and May 2024, KOA patients undergoing treatment at our hospital and healthy individuals who received physical assessments in the same timeframe were included. Approval for this research project has been granted by the Ethics Committee, and each enrolled individual has provided signed informed consent. Sample size estimation: A prospective cohort design was adopted, with suboptimal rehabilitation as the primary outcome. This outcome was defined as either a <30% improvement in the Western Ontario and McMaster Universities Osteoarthritis Index (WOMAC) score compared to baseline or an inability to achieve a ≥2-point reduction in the Visual Analogue Scale (VAS) pain score at the 12-month follow-up. Using pilot study data (a small sample of 20 people, suboptimal rehabilitation incidence: 28%, HR=2.5) and analogous studies [10], the Cox proportional hazards model was applied to calculate the required sample size. With α set at 0.05 (two-sided) and β at 0.2 (80% statistical power), 162 cases were needed for each group. A 10% loss-to-follow-up rate was factored in, resulting in a final sample size of 180 cases. The sample size was calculated using PASS15.0 software.

### Case selection criteria

Inclusions: (1) Clinical KOA diagnosis [11]; (2) Kellgren-Lawrence (KL) grade ≥ 2 [12]; (3) Age 40-80 years; (4) Symptom duration ≥ 6 months; (5) Baseline WOMAC score ≥ 30 [13]; (6) Signed informed consent.

Exclusions: (1) Secondary arthritis (rheumatoid, gouty, etc.); (2) Recent (3-month) use of bone-active drugs (bisphosphonates, calcitonin, glucocorticoids) or immunosuppressants; (3) Severe cardiac, hepatic, or renal comorbidity (estimated glomerular filtration rate [eGFR] < 30 mL/min/1.73 m^2^; alanine transaminase [ALT]/aspartate transaminase [AST] > 2 × upper limit of normal); (4) Pregnancy or lactation; (5) Psychiatric/cognitive disorders impeding compliance; (6) Knee infections, tumors, or severe deformities.

### Grouping and follow-up protocol

This prospective cohort study included 184 KOA cases and 180 baseline-matched (age, gender, etc.) healthy controls. Upon hospital admission, attending physicians developed personalized treatment plans for each KOA patient based on disease severity to ensure therapeutic consistency across the cohort. Moreover, all KOA patients were subjected to a 1-year prognostic follow-up, during which the rate of unfavorable rehabilitation at the 1-year mark was documented.

### Laboratory indexes

Fasting venous blood was obtained from all participants at admission. Following collection, samples were promptly centrifuged (3000 × g, 10 min) to prevent interference from hemolysis or lipemia. The resulting serum was then distributed into aliquots in EP tubes, with careful attention to restrict repeated freeze-thaw cycles to three or fewer. An ELISA methodology was employed to determine serum PINP CTX, PGE2, NE, IL-1β, IL-6, and TNF-α contents. Specifically, the assay required pipetting 50 μL of serum into wells, incubating with 200 μL of enzyme-labeled antibody for two hours at room temperature, and subsequently washing the plate five times (1-minute soak per wash). Following Tetramethylbenzidine (TMB) substrate addition and a 15-minute incubation, the reaction was terminated using 2 mol/L H_2_SO_4_, after which absorbance was read at 450 nm with the help of a microplate reader. PINP concentration was calculated based on a standard curve. For quality assurance, each batch included low (20 ng/mL), medium (50 ng/mL), and high (100 ng/mL) concentration controls (Bio-Rad). Any assay with an intra-batch coefficient of variation (CV) exceeding 5% was repeated.

For radioimmunoassay-based detection of SP [SP quantification utilized RIA due to its superior sensitivity (detection limit 0.1 pg/mL) for low-concentration neuropeptides compared to ELISA], serum samples underwent extraction with ethyl acetate, and the extracted fraction was dried in a vacuum environment. After drying, ^125^I-SP and anti-SP antibody were introduced, followed by an 18-hour incubation period at 4°C. Immune complexes were then precipitated using a secondary antibody, and the level of radioactivity was measured with a gamma counter. Quality control measures consisted of maintaining non-specific binding (NSB) below 5% and the inclusion of zero-standard wells (background controls) in each run.

Laboratory personnel were blinded to the group assignment (KOA patient or healthy control) and rehabilitation outcome status during biomarker assays.

### Statistical analysis

Data processing was performed using SPSS 30.0 software. All categorical data comparisons were carried out with the chi-square test. The distribution of continuous variables was first examined using the Shapiro-Wilk test. Based on the normality outcome, either the independent samples t-test (for normal distribution) or the Mann-Whitney U test (for non-normal distribution) was employed. Pearson correlation and receiver operating characteristic (ROC) analyses assessed associations and diagnostic utility, respectively. For multiple comparisons involving the biomarker levels, Bonferroni correction was applied. A significance threshold of P<0.05 was adopted.

## Results

### Comparison of demographic features

After statistical assessment, we identified no statistical disparities in such demographic variables as age, sex, and family history of disease between KOA patients and healthy controls (P>0.05, Table 1). This finding verified group comparability.

**Table 1:** Clinical baseline data.

Group	Age	Sex	Combined with<br>diabetes mellitus	Combined with<br>hypertension	Family history<br>of KOA
		Male vs. female	yes vs. no	yes vs. no	yes vs. no
Control (n = 180)	62.16±8.63	86 vs. 94	92 vs. 88	59 vs. 121	22 vs. 158
KOA (n = 184)	63.26±6.60	82 vs. 102	105 vs. 79	65 vs. 119	28 vs. 156
t or χ^2^	1.375	0.378	1.299	0.263	0.689
P	0.170	0.539	0.254	0.608	0.407

### Disparities in bone metabolism markers, pain mediators, and inflammatory cytokines between KOA cases and controls

**Figure 1:**
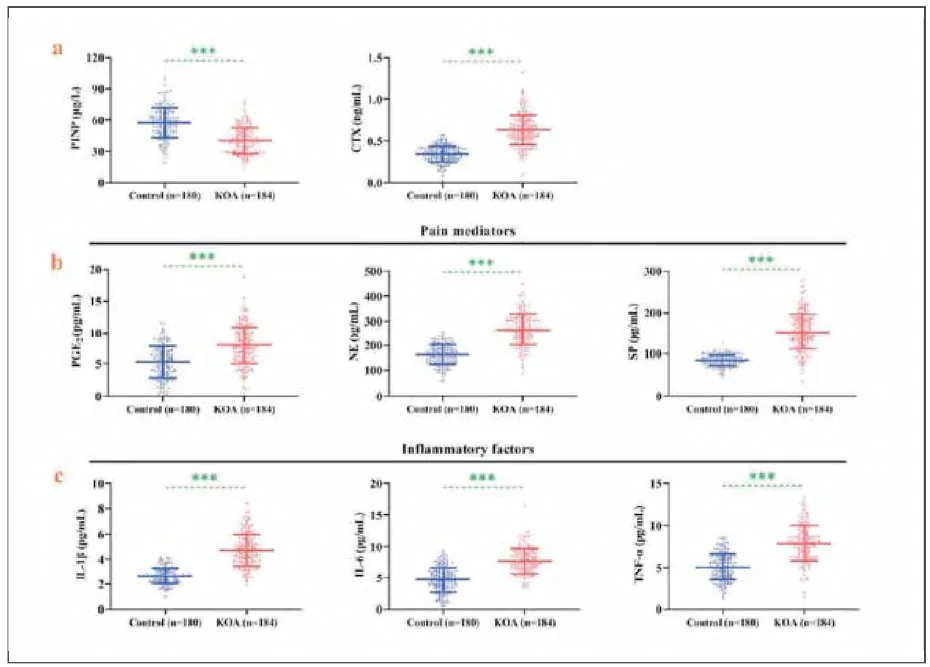
Comparison of bone metabolism, pain mediators, and inflammatory factors.

In contrast to the control group, KOA patients had lower PINP and higher CTX concentrations (P<0.05). Furthermore, KOA cases exhibited marked elevations in pain-related mediators (PGE_2_, NE, SP) and inflammatory cytokines (IL-1β, IL-6, TNF-α) than controls (P<0.05, Figure 1).

### Correlation of bone metabolism markers with pin mediators and inflammatory cytokines in KOA

**Figure 2:**
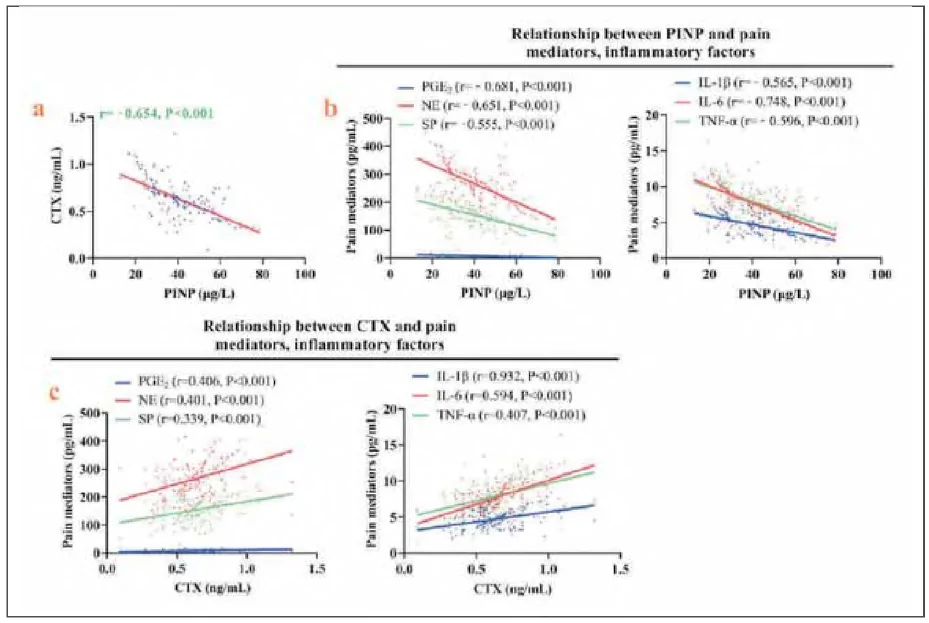
Relationship between bone metabolism, pain mediators, and inflammatory factors in KOA patients. (a) Correlation between PINP and CTX. (b) Correlation between PINP and pain mediators/inflammatory factors. (c) Correlation between CTX and pain mediators/inflammatory factors.

Analysis revealed an inverse relationship between PINP (a marker of bone formation) and the concentrations of multiple pro-inflammatory cytokines [IL-1β (r=-0.565), IL-6 (r=-0.748), TNF-α (r=-0.596)] and pain mediators [PGE2 (r=-0.681), NE (r=-0.651), SP (r=-0.555)] in KOA patients. This indicates that lower PINP concentrations are associated with higher levels of these mediators. Conversely, the bone resorption marker CTX demonstrated a positive correlation with the same set of factors [IL-1β (r=0.932), IL-6 (r=0.594), TNF-α (r=0.407), PGE2 (r=0.406), NE (r=0.401), SP (r=0.339)], meaning that lower CTX levels are linked to reduced expression of pain and inflammatory markers (Figure 2).

### Bone metabolic activity and its association with adverse rehabilitation outcomes in KOA

**Figure 3:**
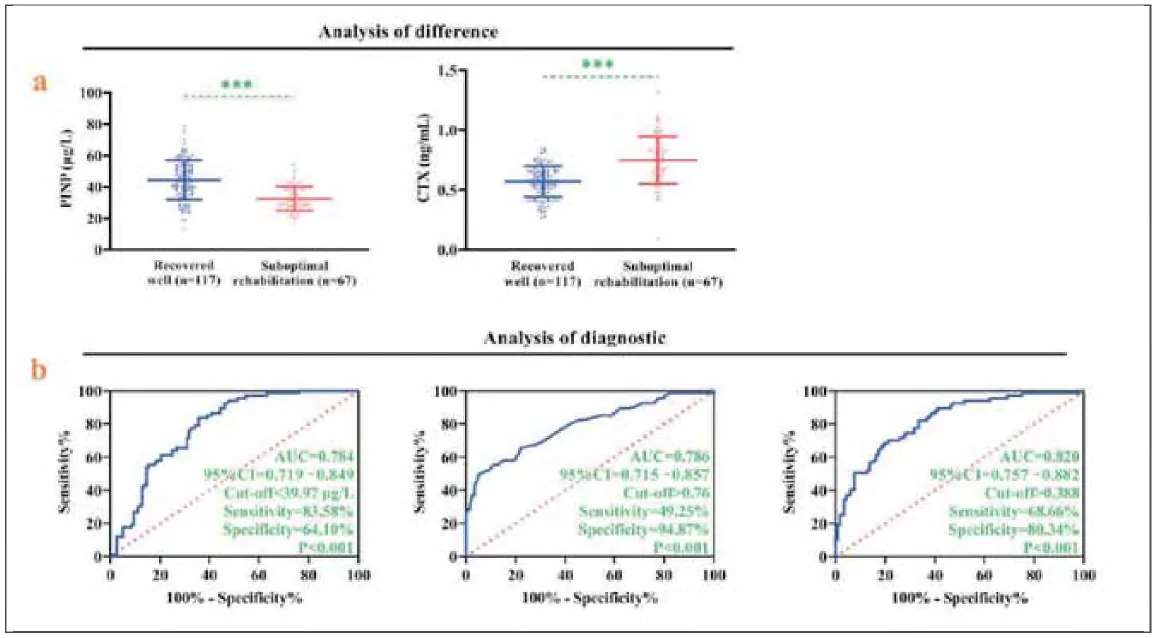
Relationship between bone metabolism and poor rehabilitation in KOA prognosis. (a) Comparison of the bone metabolic markers PINP and CTX. (b) Diagnostic value of bone metabolic markers for poor prognosis and rehabilitation in KOA. *** indicates P<0.001 for comparison between the two groups.

All patients successfully completed the follow-up. During the follow-up period, 67 cases were identified with suboptimal rehabilitation outcomes. Comparative results indicated markedly decreased PINP and elevated CTX levels in these patients relative to those who recovered well (P<0.05). The combined use of PINP and CTX showed 68.66% sensitivity and 80.34% specificity in predicting poor rehabilitation outcomes based on ROC analysis, with an AUC value of 0.820, higher than that of either biomarker alone (Figure 3).

### Development and validation of a risk assessment model for suboptimal rehabilitation outcomes in KOA based on bone metabolic, pain, and inflammatory biomarkers

**Figure 4:**
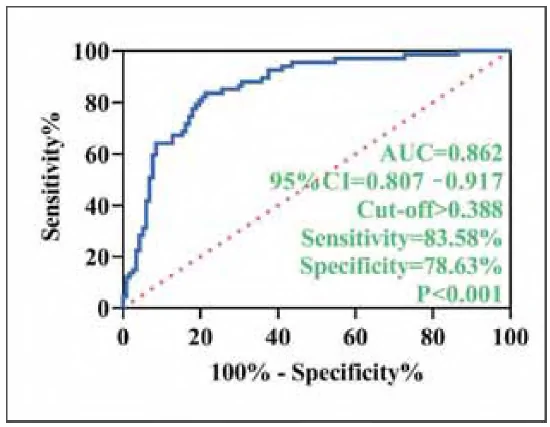
Diagnostic effect of the combined detection model of bone metabolism, pain mediators, and inflammatory factors on the prognosis of KOA with poor rehabilitation.

Similarly, comparing patients' pain mediators and inflammatory factors revealed that patients with favorable recovery exhibited significantly lower levels of PGE_2_ NE, SP IL-1β, IL-6, and TNF-α compared to those with unfavorable recovery (P<0.05, Table 2). Finally, we assessed the levels of bone metabolic, pain-associated, and inflammatory biomarkers among patients with varying rehabilitation outcomes and built a multivariate logistic regression model (forward stepwise method). The output results showed that NE, SP IL-1β and TNF-α were not independent factors affecting the poor rehabilitation of KOA (P >0.05), while PINP, pGe_2_ and IL-6 were independent factors (P<0.05, Table 3). Subsequently, after controlling for non-independent factors, we established a combined risk model based on regression analysis results and validated its effectiveness. An integrative risk model based on these biomarkers (PINP CTX, PGE2, and IL-6) exhibited high predictive accuracy, achieving 83.58% sensitivity, 78.63% specificity, and an AUC of 0.862, which underscores its potential clinical utility (Figure 4).

**Table 2:** Comparison of pain mediators and inflammatory factors.

Groups	PGE_2_ (pg/mL)	NE (pg/mL)	SP (pg/mL)	IL-1β (pg/mL)	IL-6 (pg/mL)	TNF-α (pg/mL)
Recovered well (n=117)	7.57±3.24	257.08±63.64	149.23±45.47	4.40±1.05	7.27±1.72	7.55±1.63
Suboptimal rehabilitation<br>(n = 67)	8.66±2.36	281.56±60.15	162.39±37.17	5.25±1.40	8.43±2.18	8.52±2.67
t	2.415	2.561	2.014	4.609	3.985	3.079
P	0.017	0.011	0.046	<0.001	<0.001	0.003

**Table 3:** Multivariate analysis of factors influencing the prognosis of KOA with poor rehabilitation.

	B	SE	Wald	OR	95%CI		P
					Lower limit	Upper limit	
PINP	-0.201	0.046	19.533	0.818	0.748	0.894	<0.001
CTX	6.161	1.874	10.805	1.704	1.027	1.851	0.001
PGE_2_	-0.296	0.11	7.274	0.744	0.6	0.922	0.007
NE	-0.009	0.005	3.64	0.991	0.981	1.000	0.056
SP	-0.004	0.006	0.532	0.996	0.984	1.007	0.466
IL-1β	0.291	0.194	2.243	1.338	0.914	1.958	0.134
IL-6	-0.399	0.179	4.947	0.671	0.472	0.954	0.026
TNF-α	-0.089	0.131	0.466	0.914	0.707	1.182	0.495

## Discussion

As the most common degenerative joint disease in the world, the core case mechanism of KOA is cartilage degeneration [14]. Emerging evidence has indicated the role of bone metabolism imbalance (featuring inhibited bone formation and enhanced bone resorption) as a crucial driving force for KOA progression and a contributor to clinical symptom exacerbation by regulating pain mediators and inflammatory cytokines [15]. This study is the first to systematically explore the correlation of bone metabolism markers with serum pain mediators (PGE2, NE, SP) and inflammatory cytokines (IL-1β, IL-6, TNF-α) in KOA. Furthermore, a multi-biomarker-based prediction model for unfavorable rehabilitation outcomes was developed. The results showed notably reduced levels of PINP a bone formation marker, as well as markedly increased levels of CTX (a bone resorption marker), pain mediators, and inflammatory cytokines, in KOA cases versus controls. The AUC of the prediction model constructed by combining PINP CTX, PGE_2_, and IL-1β for suboptimal rehabilitation outcomes reached 0.862, with a sensitivity of 83.58% and a specificity of 78.63%, which was significantly better than that of a single indicator.

The interplay between abnormal bone metabolism, pain mediators, and inflammatory cytokines constitutes the core link of the complex pathological network of KOA. This study observed an inverse correlation between the bone formation marker PINP and levels of both pain mediators and inflammatory markers, suggesting that inhibited bone formation may exacerbate disease progression by activating the inflammation-pain axis. These findings align with existing literature, which shows that osteoprotegerin (OPG), secreted by osteoblasts, inhibits osteoclast activation by antagonizing the receptor activator of nuclear factor- B ligand (RANKL) signal, while OPG deficiency can lead to enhanced bone resorption and synovial inflammation [16]. Furthermore, the inverse regulatory relationship observed between PINP levels and inflammatory markers may be linked to a pro-inflammatory milieu facilitated by osteoblast dysfunction. This decline in osteoblast activity not only reduces the secretion of anti-inflammatory mediators like IL-10 but also facilitates a shift toward M1 macrophage polarization, further amplifying the expression of pro-inflammatory cytokines including IL-6 and TNF-α [17]. Conversely, the positive association of the bone resorption indicator CTX with both pain-related molecules and inflammatory markers highlights how increased bone resorption may directly influence pain signaling pathways. Cytokines released during osteoclast activation are known to stimulate cyclooxygenase-2 (COX-2) expression in synovial fibroblasts, which in turn catalyzes the synthesis of PGE_2_ [18]. Through its action on EP2/EP4 receptors, PGE_2_ then exerts a dual effect: it both activates peripheral nociceptive nerve endings and stimulates glial cells to release neurotransmitters like glutamate. These processes collectively establish a vicious feedback loop integrating bone resorption, inflammation, and pain sensation [19].

Subsequent analysis confirmed the significant predictive value of baseline bone metabolism levels for the one-year rehabilitation outcomes in KOA patients. Subsequently, ROC analysis indicated a markedly higher AUC for the PINP+CTX combination (0.820) than for either marker alone, highlighting the predictive complementarity of bone metabolism markers within a predictive framework. The predictive power was further enhanced by integrating these markers with pain mediators and inflammatory cytokines into a multivariate logistic regression model. The model we built achieved a substantially improved AUC of 0.862, with 83.58% sensitivity and 78.63% specificity, indicating strong clinical translational potential. This model's advantages are dual: First, by integrating multi-dimensional indicators of "bone metabolism-pain-inflammation", it breaks through the limitations of traditional imaging evaluation and facilitates earlier identification of high-risk groups. Second, its composition of readily obtainable serum biomarkers allows for non-invasive and repeated sampling, greatly enhancing the feasibility of dynamic tracking and personalized care. The model's emphasis on inflammatory cytokines, in particular, validates targeting inflammation (e.g., IL-1 receptor antagonists) as a key component of rehabilitation, opening new doors for precise clinical management.

We propose that in the future, PINP and CTX could be incorporated into the standard monitoring protocol for KOA patients. Integrating these markers with the WOMAC score would allow for dynamic assessment of disease progression and therapeutic response. It is important to note, however, that while this study offers valuable insights, it has certain limitations. The single-center design and a sample size of 180 patients may introduce bias, necessitating validation through larger, multi-center cohorts. Additionally, while this study identifies associations, its cross-sectional design cannot establish causation between bone metabolic dysregulation and pain mediator levels. Future work must therefore employ longitudinal data or animal-based intervention experiments to confirm a causal mechanism.

## Conclusion

This study demonstrates that dysregulated bone metabolism (low PINP, high CTX) in KOA correlates significantly with elevated pain mediators and inflammatory cytokines. A combined biomarker panel (PINP, CTX, PGE2, IL-1β) effectively predicts patients at risk of poor rehabilitation outcomes. These findings advance our understanding of the bone-pain-inflammation interplay in KOA and provide a foundation for personalized risk stratification and targeted management strategies.

## Dodatak

### Availability of data and materials

The data that support the findings of this study are available from the corresponding author upon reasonable request.

### Conflict of interest statement

All the authors declare that they have no conflict of interest in this work.

## References

[1] Giorgino R, Albano D, Fusco S, Peretti G M, Mangiavini L, Messina C (2023). Knee Osteoarthritis: Epidemiology, Pathogenesis, and Mesenchymal Stem Cells: What Else Is New? An Update. Int J Mol Sci.

[2] Li M, Xia Q, Nie Q, Ding L, Huang Z, Jiang Z (2025). Burden of knee osteoarthritis in China and globally: 1990-2045. BMC Musculoskelet Disord.

[3] Mohamed S H P, Alatawi S F (2023). Effectiveness of Kinesio taping and conventional physical therapy in the management of knee osteoarthritis: A randomized clinical trial. Ir J Med Sci.

[4] Hu N, Zhang J, Wang J, Wang P, Wang J, Qiang Y, Li Z, Wu T, Wang X, Wang Y, Li J, Liu X, Zhang J, Feng X, Ju B, Hao Z, Pu D, Lu X, Wang Q, He L (2022). Biomarkers of joint metabolism and bone mineral density are associated with early knee osteoarthritis in premenopausal females. Clin Rheumatol.

[5] Chen W, Xiao J, Zhou Y, Liu W, Jian J, Yang J, Chen B, Ye Z, Liu J, Xu X, Jiang T, Wang H, Liu W (2024). Curcumenol regulates Histone H3K27me3 demethylases KDM6B affecting Succinic acid metabolism to alleviate cartilage degeneration in knee osteoarthritis. Phytomedicine.

[6] Kuroo A, Murata K, Morishita Y, Onitsuka K, Oka Y, Tanaka K, Kanemura N (2025). Controlling Joint Instability Reduces Inflammatory Pain in Early Knee Osteoarthritis. Cureus.

[7] Park E H, Seo J, Lee Y, Park K, Kim K, Kim S, Mobasheri A, Choi H (2023). TissueGene-C induces long-term analgesic effects through regulation of pain mediators and neuronal sensitization in a rat monoiodoacetate-induced model of osteoarthritis pain. Osteoarthritis Cartilage.

[8] Zhang J, Li K, Qiu X (2024). Exploring causal correlations between inflammatory cytokines and knee osteoarthritis: A two-sample Mendelian randomization. Front Immunol.

[9] Sang W, Xue S, Jiang Y, Lu H, Zhu L, Wang C, Ma J (2021). METTL3 involves the progression of osteoarthritis probably by affecting ECM degradation and regulating the inflammatory response. Life Sci.

[10] Mccabe P G, Lisboa P, Baltzopoulos B, Olier I (2022). Externally validated models for first diagnosis and risk of progression of knee osteoarthritis. PLoS One.

[11] Gibbs A J, Gray B, Wallis J A, Taylor N F, Kemp J L, Hunter D J, Barton C J (2023). Recommendations for the management of hip and knee osteoarthritis: A systematic review of clinical practice guidelines. Osteoarthritis Cartilage.

[12] Goh G S, Schwartz A M, Friend J K, Grace T R, Wickes C, Bolognesi M P, Austin M S (2023). Patients Who Have Kellgren-Lawrence Grade 3 and 4 Osteoarthritis Benefit Equally From Total Knee Arthroplasty. J Arthroplasty.

[13] Namireddy S R, Gill S S, Yaqub Y, Ramkumar P (2024). Computerized Versus Traditional Approaches for Total Knee Arthroplasty: A Quantitative Analysis of Knee Society Score and Western Ontario and McMaster Universities Osteoarthritis Index. Orthop Surg.

[14] Lee Y, Park Y S, Choi N Y, Kim Y I, Koh Y G (2021). Proteomic Analysis Reveals Commonly Secreted Proteins of Mesenchymal Stem Cells Derived from Bone Marrow, Adipose Tissue, and Synovial Membrane to Show Potential for Cartilage Regeneration in Knee Osteoarthritis. Stem Cells Int.

[15] Xie J, Wang Y, Lu L, Liu L, Yu X, Pei F (2021). Cellular senescence in knee osteoarthritis: molecular mechanisms and therapeutic implications. Ageing Res Rev.

[16] Mostafa R E, Salama A A A (2023). Eplerenone modulates the inflammatory response in monosodium iodoacetate-induced knee osteoarthritis in rats: Involvement of RANKL/OPG axis. Life Sci.

[17] Yoshida S, Nishitani K, Yoshitomi H, Kuriyama S, Nakamura S, Fujii T, Saito M, Kobori Y, Murakami A, Murata K, Ito H, Ueno H, Matsuda S (2023). Knee Alignment Correction by High Tibial Osteotomy Reduces Symptoms and Synovial Inflammation in Knee Osteoarthritis Accompanied by Macrophage Phenotypic Change From M1 to M2. Arthritis Rheumatol.

[18] Zhang C, Li X, Wen P, Li Y (2024). Ellagic acid improves osteoarthritis by inhibiting PGE2 production in M1 macrophages via targeting PTGS2. Clin Exp Pharmacol Physiol Suppl.

[19] Xiang J, Zhang Y, Wu J, Shui Y, Zhu Y (2024). Electroacupuncture combined with continuous adductor canal block for postoperative analgesia in patients undergoing total knee arthroplasty. Zhongguo zhen jiu / Chinese Acupuncture & Moxibustion.

